# First spermatological study in the Atractotrematidae (Digenea, Haploporoidea): the case of *Atractotrema sigani*, intestinal parasite of *Siganus lineatus*

**DOI:** 10.1051/parasite/2015026

**Published:** 2015-10-16

**Authors:** Abdoulaye J. S. Bakhoum, Yann Quilichini, Jean-Lou Justine, Rodney A. Bray, Jordi Miquel, Carlos Feliu, Cheikh T. Bâ, Bernard Marchand

**Affiliations:** 1 CNRS-Università di Corsica, UMR 6134-SPE, SERME Service d’Étude et de Recherche en Microscopie Électronique Corte 20250 Corsica France; 2 Laboratory of Evolutionary Biology, Ecology and Management of Ecosystems, Cheikh Anta Diop University of Dakar BP 5055 Dakar Senegal; 3 ISYEB, Institut de Systématique, Évolution, Biodiversité (UMR7205 CNRS, EPHE, MNHN, UPMC), Muséum National d’Histoire Naturelle, Sorbonne Universités CP 51 55 rue Buffon 75231 Paris cedex 05 France; 4 Department of Life Sciences, Natural History Museum Cromwell Road SW7 5BD London UK; 5 Laboratori de Parasitologia, Departament de Microbiologia i Parasitologia Sanitàries, Facultat de Farmàcia, Universitat de Barcelona Av. Joan XXIII, sn 08028 Barcelona Spain; 6 Institut de Recerca de la Biodiversitat, Facultat de Biologia, Universitat de Barcelona Av. Diagonal 645 08028 Barcelona Spain

**Keywords:** Cell Biology, Platyhelminthes, Digenea, Ultrastructure, Spermatozoon, Phylogeny

## Abstract

The ultrastructural organization of the mature spermatozoon of the digenean *Atractotrema sigani* (from *Siganus lineatus* off New Caledonia) was investigated by transmission electron microscopy. The male gamete of *A. sigani* exhibits the general morphology described in digeneans with the presence of two axonemes of different lengths showing the 9 + “1” pattern of the Trepaxonemata, a nucleus, two mitochondria, two bundles of parallel cortical microtubules, external ornamentation, spine-like bodies and granules of glycogen. However, the mature spermatozoon of *A. sigani* has some specific characters such as the morphology of its anterior region and the submembranous electron-dense material. Although similar structures have been reported in some digenean species, the presence of a submembranous electron-dense material describing a complete ring is reported here for the first time in the mature spermatozoon of *A. sigani*. In addition, sperm characteristics are compared between the Haploporoidea and their supposed close superfamilies, and possible phylogenetic implications of these findings for the Digenea are discussed.

## Abstract (fr)

L’organisation ultrastructurale du spermatozoïde mûr du digène *Atractotrema sigani* (parasite de *Siganus lineatus* en Nouvelle-Calédonie) est décrite au microscope électronique à transmission. Le gamète mâle d’*A. sigani* présente la morphologie générale des spermatozoïdes décrits chez les digènes avec la présence de deux axonèmes de type 9 + “1” des Trepaxonemata, un noyau, deux mitochondries, deux champs de microtubules corticaux parallèles, une ornementation externe, des corps épineux et des granules de glycogène. Cependant, le spermatozoïde mûr d’*A. sigani* présente certains caractères spécifiques tels que la morphologie de son extrémité antérieure et le matériel sous-membranaire opaque aux électrons. Bien qu’une structure similaire à cette dernière fût décrite précédemment chez certaines espèces, la présence d’un matériel opaque aux électrons décrivant un anneau complet est observée pour la première fois dans le spermatozoïde de *A. sigani*. De plus, les caractères spermatiques sont comparés entre les Haploporoidea et les superfamilles qui sont censées leur être proches et les possibles implications phylogénétiques de ces résultats pour les Digenea sont discutées.

## Introduction

The Atractotrematidae are parasites of the intestine or gall bladder of marine or estuarine teleosts. This family includes four genera, namely *Atractotrema*, *Isorchis*, *Pseudisorchis* and *Pseudomegasolena*. Atractotrematid species are mainly characterized by the presence of two symmetrical or slightly oblique testes in the hindbody or forebody, vitelline fields with follicles often interconnected as elongate lobes, a hermaphroditic sac enclosing both male and female ducts and a Y-shaped excretory vesicle [37].

The systematic position and taxonomy of the Atractotrematidae still offer challenges at several levels. Previously, the family was considered to be close to the Fellodistomidae [48] or as a synonym of the Haploporidae [1]. However, on the basis of morphological similarities and recent molecular findings, the Atractotrematidae was proposed as a distinct family, closely related to the Haploporidae with which they form the superfamily Haploporoidea [12, 14, 36].

The above historical account indicates the need for a phylogenetic analysis based on an additional, independent set of characters in order to clarify the digenean phylogenetic affinities. In this sense, several authors have proposed ultrastructural characteristics of the mature spermatozoon in digeneans, as is the case in cestodes and monogeneans (the other neodermatan group widely studied) [6, 8, 19–21, 25, 31, 33].

Spermatological characteristics available in the Haploporoidea concern only the haploporid *Saccocoelioides godoyi* [11]. In the present study, we describe for the first time the ultrastructure of the male gamete of *Atractotrema sigani* belonging to the Atractotrematidae.

## Materials and methods

Adult specimens of *Atractotrema sigani* Durio and Manter, 1969 were obtained from the digestive tract of *Siganus lineatus* from the fish market of Nouméa, New Caledonia, South Pacific, with the wash method [22]. Voucher specimens were mounted on slides and are kept in the collections of the Muséum National d’Histoire Naturelle, Paris (as MNHN JNC2873) and Natural History Museum, London. For ultrastructural studies, adult digeneans were fixed in cold (4 °C) 2.5% glutaraldehyde in 0.1 M sodium cacodylate buffer at pH 7.2, rinsed in 0.1 M sodium cacodylate buffer at pH 7.2, post-fixed in cold (4 °C) 1% osmium tetroxide in the same buffer for 1 h, dehydrated in ethanol and propylene oxide series, embedded in Spurr resin and polymerized at 60 °C for 24 h. Ultrathin sections (60–90 nm) of the seminal vesicle were cut on an ultramicrotome (Power tome PC, RMC Boeckeler^®^). The sections were placed on 200 and 300 mesh copper grids and stained with uranyl acetate and lead citrate according to Reynolds’ methodology [44]. The cytochemical technique established by Thiéry [47] was also used to locate glycogen in sections placed on gold grids. Finally, all grids were examined on a Hitachi H-7650 transmission electron microscope, operating at an accelerating voltage of 80 kV, in the “Service d’Étude et de Recherche en Microscopie Électronique” of the University of Corsica Pasquale Paoli (Corte, France).

## Results

An analysis of several cross- and longitudinal sections from the seminal receptacle of *A. sigani* has allowed the reconstitution of the mature spermatozoon from the anterior to the posterior extremity. Thus, five regions (I–V) exhibiting different ultrastructural characteristics were distinguished in the male gamete of *Atractotrema sigani*.

**Figure 1.**
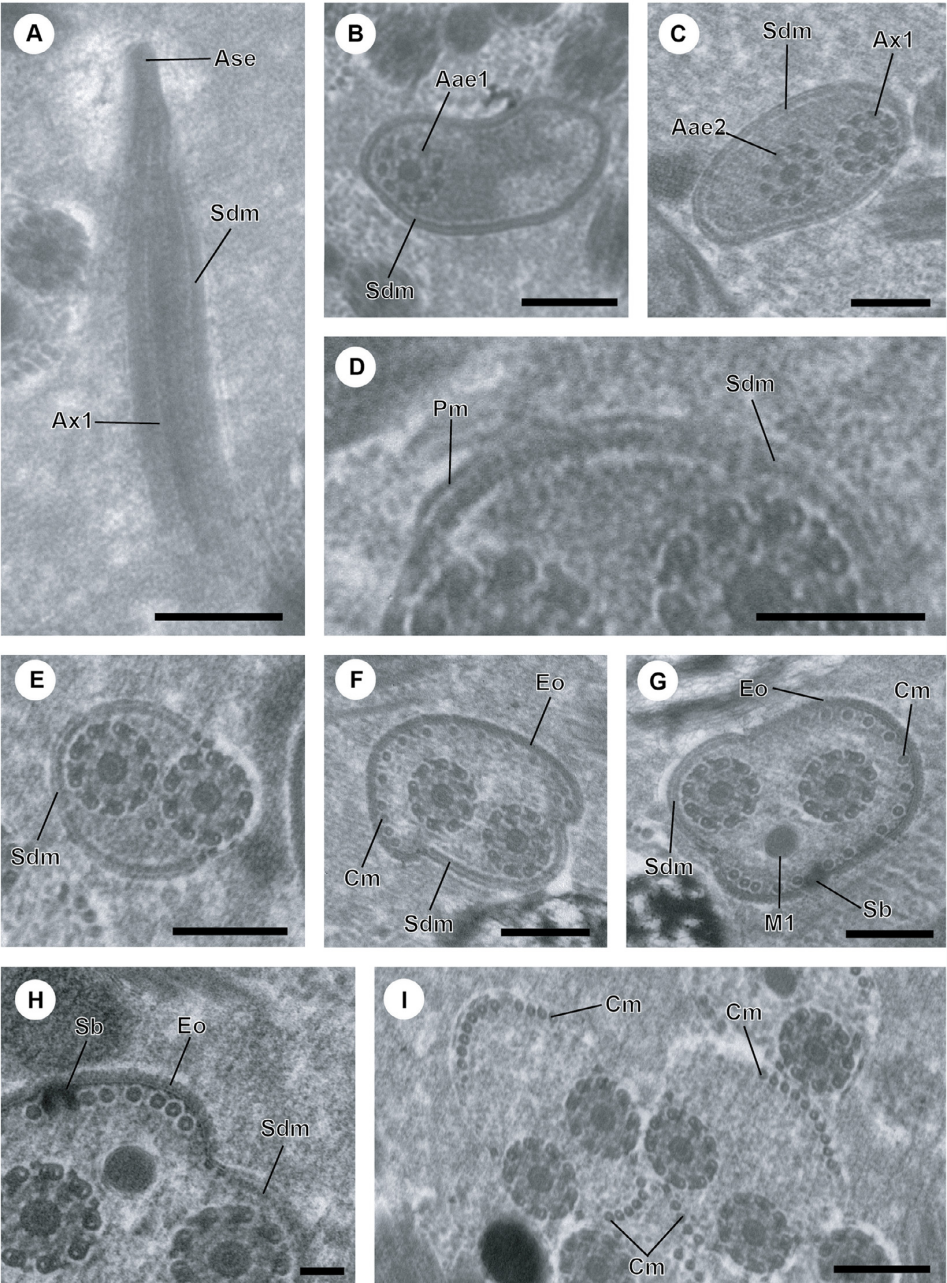
A–I. Mature spermatozoon of *Atractotrema sigani* in regions I–III. (A) Longitudinal section of anterior spermatozoon extremity (Ase) with a sharp tip. Sdm, submembranous electron-dense material; Ax1 first axoneme. (B) Cross-section showing the anterior extremity of the first axoneme (Aae1) and the submembranous electron-dense material describing a complete ring. (C) Cross-section in region I exhibiting the first axoneme and the anterior extremity of the second axoneme (Aae2). Note also the submembranous electron-dense material surrounding both axonemes. (D) Detail of submembranous electron-dense material of *A. sigani*. Pm, plasma membrane; Sdm, submembranous electron-dense material. (E) Cross-section in posterior part of region I with the axonemes and the submembranous electron-dense material located laterally. (F, G) Consecutive cross-sections in region II characterized by the presence of external ornamentation of the plasma membrane (Eo), the first mitochondrion (M1) and spine-like body (Sb). Note the cortical microtubules (Cm) associated with the external ornamentation and the residual submembranous electron-dense material not associated with cortical microtubules. (H) Detail showing the simultaneous presence of external ornamentation, spine-like body and submembranous electron-dense material. (I) Cross-section in the transitional area prior to the nuclear region, showing the axonemes and cortical microtubules distributed into two fields and their maximum number varies between 14 and 17. Scale in μm: (A, B, C, E, F, G, I), 0.3; (D), 0.2; (H), 0.1.

Region I (Figs. 1A–E and 4I) constitutes the anterior part of the spermatozoon showing in longitudinal section a sharp tip (Fig. 1A). In cross-section, the anterior spermatozoon extremity is characterized by the presence of the anterior extremity of the first axoneme and the submembranous electron-dense material which forms a complete ring (Figs. 1A and 4I). Consecutive cross-sections towards the posterior part of region I show the anterior extremity of the second axoneme accompanied by the first axoneme and the submembranous electron-dense material. This latter is reduced and located laterally when both axonemes are completely formed (Figs. 1C–E and 4I).

**Figure 2.**
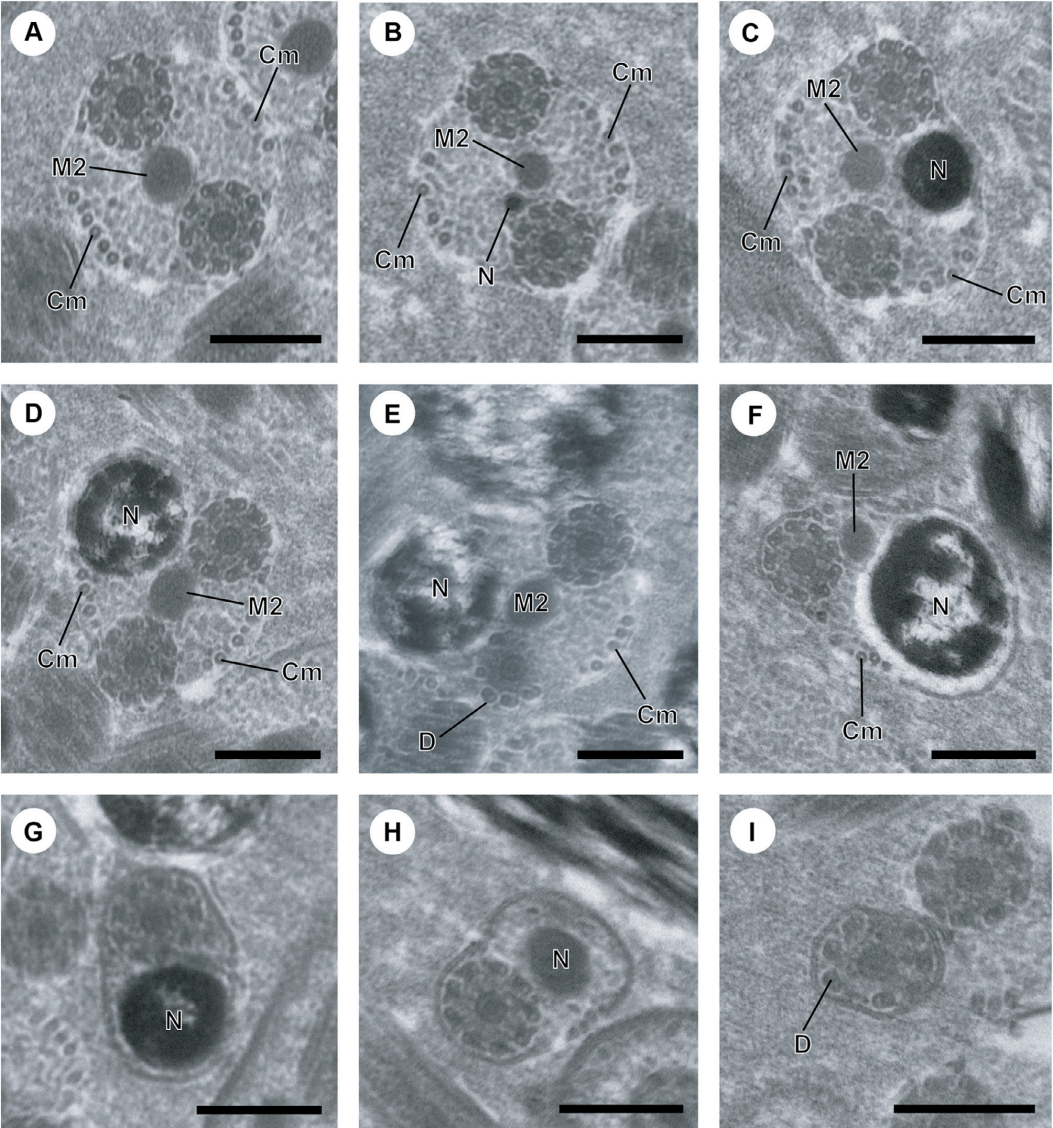
A–I. Mature spermatozoon of *Atractotrema sigani* in regions IV–V. (A) Cross-section in proximal part of region IV exhibiting the second mitochondrion (M2). Cm, cortical microtubules. Note the decrease in the maximum number of cortical microtubules (about 9) compared to region III. (B–D) Consecutive cross-sections showing the simultaneous presence of the second mitochondrion and the nucleus (N) which increases progressively in size. (E) Disorganization of the first axoneme resulting into doublets of microtubules. D, doublet of microtubules. (F) Distal part of region IV with only the second axoneme, the second mitochondrion and few cortical microtubules (about 4). (G, H) Cross-section showing only the second axoneme, the nucleus and its progressive reduction in size. (I) Cross-section of the posterior spermatozoon tip, where the nucleus disappears and only one axoneme is still observed. Scale in μm: (A–I), 0.3.

**Figure 3.**
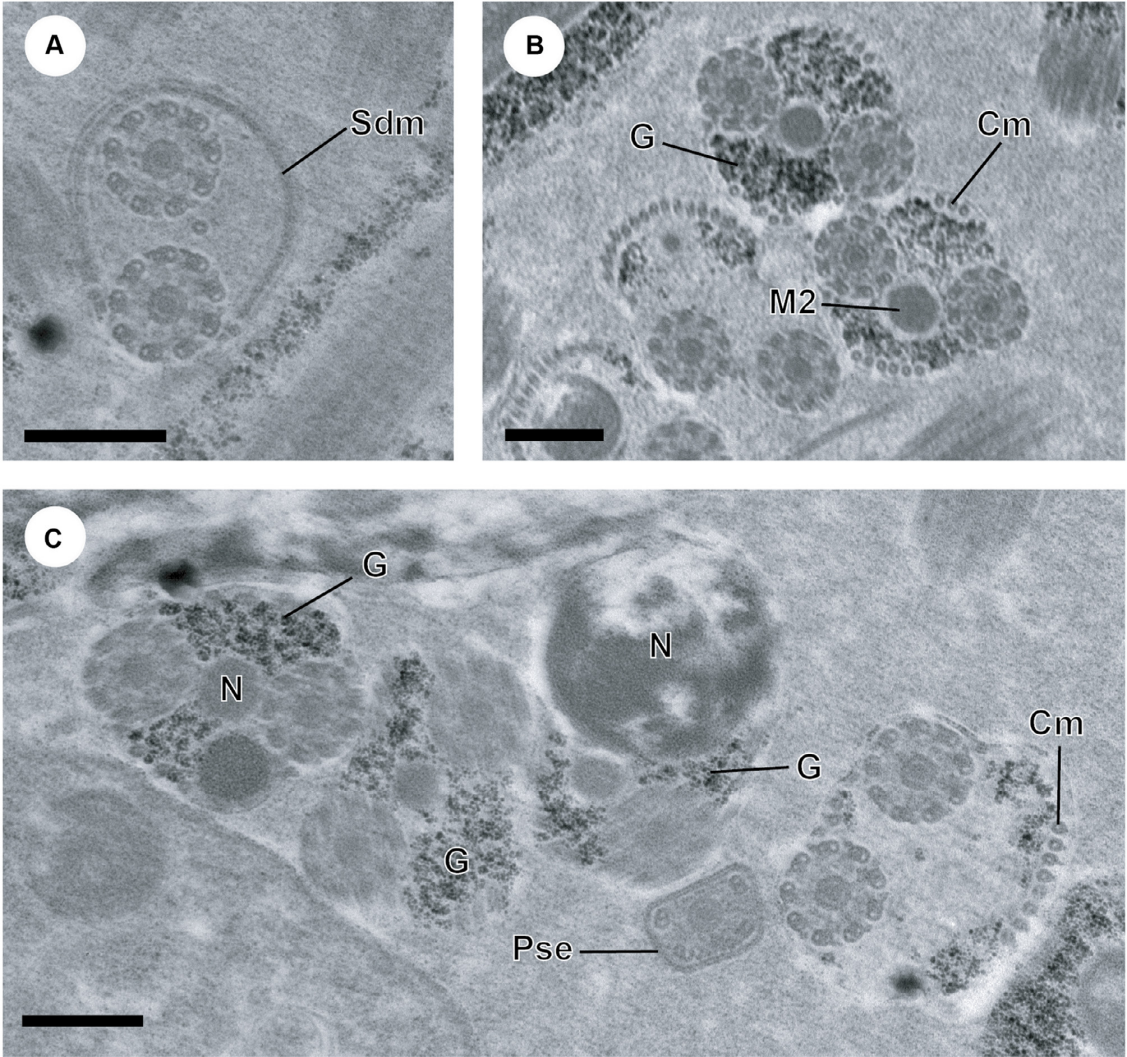
A–C. Cytochemical test of Thiéry (1967) in the spermatozoon of *Atractotrema sigani*. (A) Transmission electron micrographs showing the absence of granules of glycogen in region I. (B, C) Cross-sections exhibiting the presence of granules of glycogen in regions III–V of the mature spermatozoon of *A. sigani*. Cm, cortical microtubules; G, glycogen granules; M2, second mitochondrion; N, nucleus; Pse, posterior spermatozoon extremity. Scale in μm: (A–C), 0.3.

**Figure 4.**
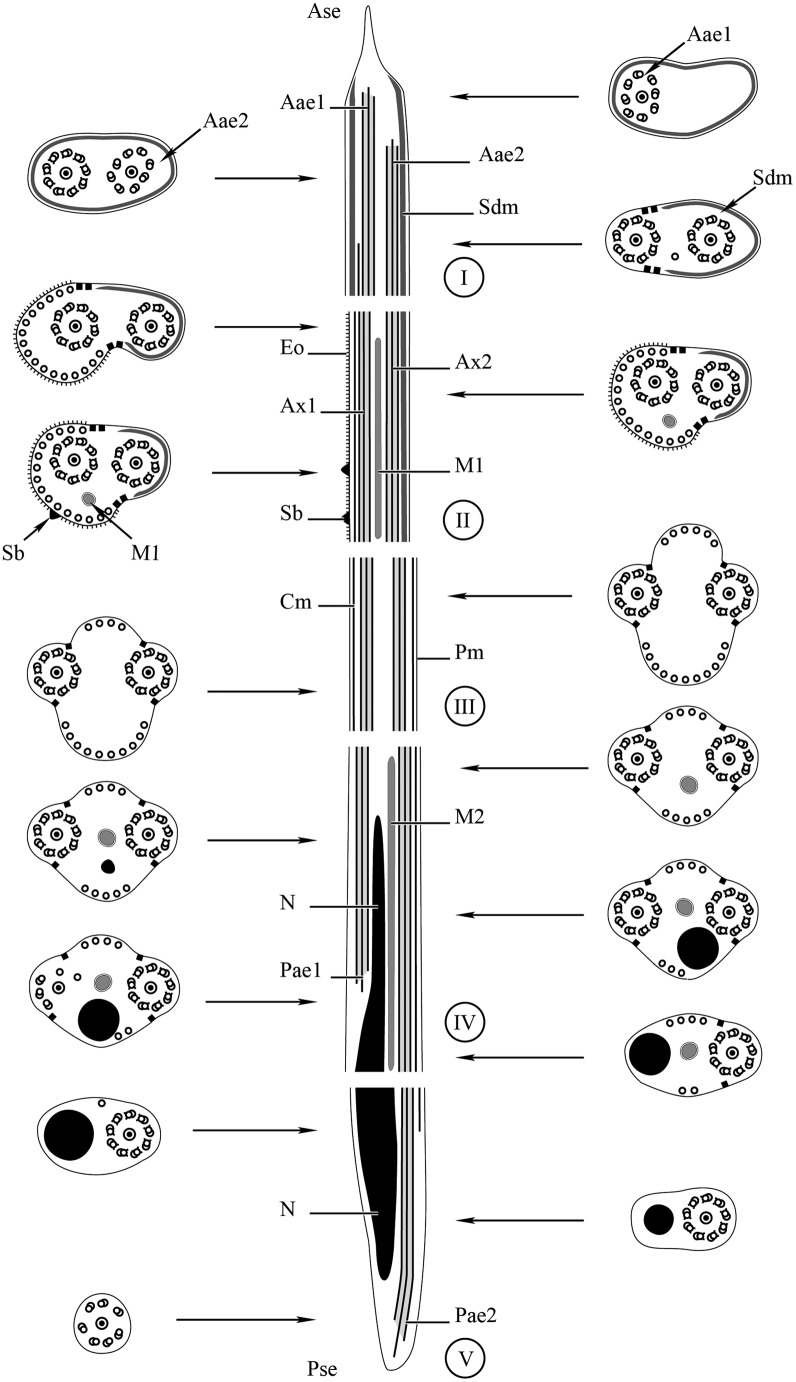
Schematic drawing of the spermatozoon of *Atractotrema sigani*. Aae1, anterior extremity of first axoneme; Aae2, anterior extremity of second axoneme; Ase, anterior spermatozoon extremity; Ax1, first axoneme; Ax2, second axoneme; Cm, cortical microtubules; Eo, external ornamentation of the plasma membrane; M1, first mitochondrion; M2, second mitochondrion; N, nucleus; Pae1, posterior extremity of axoneme 1; Pae2, posterior extremity of axoneme 2; Pm, plasma membrane; Pse, posterior spermatozoon extremity; Sb, spine-like body; Sdm, submembranous electron-dense material.

Region II (Figs. 1F–H and 4II) corresponds to the ornamented area of the mature spermatozoon, showing in several cross-sections the presence of external ornamentation of the plasma membrane, submembranous electron-dense material, the axonemes, first mitochondrion, spine-like bodies and cortical microtubules (Fig. 1F, G). The cortical microtubules (of which the maximum number is about 18) are observed on the side of the mature spermatozoon covered by the external ornamentation, whereas they are absent on the side containing the submembranous electron-dense material (Figs. 1F–H and 4II). Besides the association “cortical microtubules + external ornamentation”, it is also interesting to note the progressive reduction of submembranous electron-dense material. In addition, the external ornamentation and submembranous electron-dense material are interrupted in the posterior part of this region II.

Region III (Figs. 1I and 4III) is the transitional area before the nuclear region. It is characterized by the presence of only two axonemes and cortical microtubules separated into two fields. Considering the numerous cross-sections showing only these characters, this region appears as a large portion of the mature spermatozoon. In addition, the maximum number of cortical microtubules is reduced from 14 to 17 (Figs. 1I and 4III).

Region IV (Figs. 2A–F and 4IV) is mainly distinguished by the appearance of the second mitochondrion in its anterior part and the nucleus in its middle and posterior part. The anterior part contains only the second mitochondrion, both axonemes and cortical microtubules of which the maximum number (about 9) (Fig. 2A) is lower than in region III. When the nucleus appears in the middle part of this region IV, the axonemes, the second mitochondrial and the two fields of cortical microtubules are still present. However, the maximum number of cortical microtubules progressively diminishes from 9 (Fig. 2B), 8 (Fig. 2C) to 7 (Fig. 2D). Cross-sections in the posterior part of region IV exhibit the posterior extremity of the first axoneme with disorganized doublets of microtubules (Fig. 2E). Consequently, only one axoneme accompanied by the second mitochondrion, nucleus and few cortical microtubules (about 5) are observed (Figs. 2F and 4IV).

Region V (Figs. 2A–D and 4IV) corresponds to the posterior spermatozoon extremity. It is characterized in its proximal part by the presence of the nucleus and one axoneme (Fig. 2G). Compared to region IV, the second mitochondrion is absent. Moreover, the nucleus and cortical microtubules disappear in the proximal area of this region V. With respect to the distal part of this region, cross-section shows only one axoneme which constitutes the last ultrastructural character in the posterior tip of the mature spermatozoon.

The granules of glycogen irregularly distributed along the mature spermatozoon of *A. sigani* are evidenced by the cytochemical test of Thiéry [47] (Fig. 3A–C). It is interesting to note the absence of glycogen in the anterior spermatozoon extremity (region I) (Fig. 3A).

## Discussion

The ultrastructural organization of the mature spermatozoon of *A. sigani* is in agreement with that reported for most digenean species with the presence of two axonemes of the 9 + “1” pattern of trepaxonematans [17], nucleus, mitochondria, an association between external ornamentation of the plasma membrane and cortical microtubules, two bundles of parallel cortical microtubules generally arranged in the middle area of the spermatozoon and granules of glycogen irregularly distributed [3, 7, 16, 26, 29, 38, 40, 49].

Among the Haploporoidea, only one species, *S. godoyi*, belonging to the Haploporidae has been partially investigated for sperm ultrastructure [11]. Because of the lack of data in *S. godoyi*, no complete comparative study is attempted between its mature spermatozoon and that of *A. sigani*. However, as observed in the sperm cell of *A. sigani*, the male gamete of *S. godoyi* seems to contain two axonemes of the 9 + “1” pattern of the Trepaxonemata, two bundles of cortical microtubules [11], nucleus and mitochondria. In addition to the classical structures mentioned above, some specific characteristics distinguish the mature spermatozoon of *A. sigani* from those of other digenean taxa.

### Particularities of the spermatozoon of *A. sigani*

The ultrastructural features that characterize the spermatozoon of *A. sigani* are mainly located in the non-nuclear region, especially between the anterior tip and the end of the ornamented area. In fact, the great variety of morphologies and characters observed in the non-nuclear region in digenean spermatozoa might be of great value for phylogenetic considerations [19].

The anterior extremity of the male gamete of *A. sigani* is characterized by the presence of one axoneme (observed in longitudinal section) and a submembranous electron-dense material. In Digenea, mature spermatozoa exhibiting one axoneme in their anterior extremity are frequent and reported in several families such as the Cryptogonimidae [18, 38, 46], Gyliauchenidae [9, 40], Lepocreadiidae [7, 23, 30], Opisthorchiidae [49] or Plagiorchiidae [32, 34]. Besides the presence of one axoneme, the anterior part of the mature spermatozoon of *A. sigani* also contains submembranous electron-dense material. This latter describes a complete ring surrounding the first axoneme and appears laterally when the second axoneme is completely formed. The complete ring of submembranous electron-dense material is described here, for the first time, in the anterior extremity of the digenean spermatozoa. However, structures like submembranous electron-dense material called also “antero-lateral electron-dense material” or “electron-dense material” (according to the appellation of the authors) have been described previously in digenean spermatozoa belonging, particularly to the superfamily Lepocreadioidea *sensu* Bray and Cribb [13]. This is the case in *Holorchis micracanthum* (Aephnidiogenidae), *Gyliauchen* sp. and *Robphildollfusium fratum* (Gyliauchenidae), *Hypocreadium caputvadum*, *Neomultitestis aspidogastriformis* and *Opechona bacillaris* (Lepocreadiidae) [2, 7, 9, 23, 30, 35, 40].

An additional peculiarity that distinguishes the mature spermatozoon of *A. sigani* is the absence of cortical microtubules in its anterior extremity and in the side of the submembranous electron-dense material. In fact, the cortical microtubules appear only when both axonemes are completely formed, and they are located on the external ornamentation side.

Compared to the lepocreadioid spermatozoa reported so far, the appearance of cortical microtubules is also noted only when both axonemes are completely formed [7, 30]. However, in the mature spermatozoon of *A. sigani* the submembranous electron-dense material is still observed when cortical microtubules appear and in the ornamented region.

Another specific morphological character of the male gamete of *A. sigani* concerns the ornamented area. For the first time, the simultaneous presence of external ornamentation of the plasma membrane, cortical microtubules, spine-like bodies and submembranous electron-dense material is described in digenean mature spermatozoa. However, the association “external ornamentation + cortical microtubules + spine-like bodies” observed in the area containing the first mitochondrion has been described previously in other digenean species [5, 6, 8, 15, 24, 32, 35].

It is also interesting to notice the location of external ornamentation in the mature spermatozoon of *A. sigani*. In fact, external ornamentation of the plasma membrane appears in the anterior part (region II) of the spermatozoon, where both axonemes are already formed and accompanied by the first mitochondrion. Following the three types of digenean spermatozoa according to the location of external ornamentation established by Quilichini et al. [40], the spermatozoon of *A. sigani* exhibits type 2, i.e. presence of external ornamentation in the distal part of the anterior spermatozoon, associated with the anterior mitochondrion. Mature spermatozoa with type 2 external ornamentation have also been described so far in other digenean species [4, 7, 15, 24, 30, 35].

As stated above for the non-nuclear region, the morphology of the posterior spermatozoon extremity presents variability in digenean species and has been proposed as an interesting criterion for phylogenetic analyses [6, 29, 39]. Quilichini et al. [39] were the first to propose three types of posterior spermatozoon morphologies according to the sequence of disappearance of characters towards the posterior tip. Following this criterion, the mature spermatozoon of *A. sigani* exhibits the type 3 or cryptogonimidean type with the sequence “cortical microtubules, nucleus and axoneme”. However, considering the numerous variations in the sequences of characters towards the posterior extremity of the spermatozoon observed in digeneans and the lack of such information in several ultrastructural studies, we propose to consider only the terminal character of the male gamete. In this sense, the posterior spermatozoon extremity of *A. sigani* exhibits only one axoneme. This morphology has been observed in most digenean species belonging to the superfamilies Lepocreadioidea (for a review see [30] – Table 1), the Microphalloidea (see [29] – Table 1) or Opisthorchioidea [3, 18, 38, 46]. The other morphologies reported in digeneans concern spermatozoa with the nucleus at the posterior extremities, as observed in most species belonging to the Paramphistomoidea and Plagiorchioidea [4, 10, 32, 34, 45] and species with posterior spermatozoon tips containing cortical microtubules, as observed in the Opecoelidae and Opistholebetidae [26–28, 39, 41–43].

### Possible systematic implications

The systematic position of the Atractotrematidae is confused. Previously, this family was considered to be close to the Fellodistomidae [48]. The analyses of ultrastructural data available on both families question this classification. In fact, the morphology of the mature spermatozoon of the atractotrematid *A. sigani* (present study) is quite different to that of the fellodistomid *Tergestia acanthocephala* [24]. The major differences concern (1) the anterior spermatozoon extremity exhibiting one axoneme in *A. sigani,* whereas in *T. acanthocephala* two axonemes were reported, (2) the posterior spermatozoon extremity containing only one axoneme in *A. sigani* while it contains a nucleus in *T. acanthocephala* and (3) the presence of the submembranous electron-dense material in *A. sigani*, whereas it was absent in the mature sperm of *T. acanthocephala* [24].

More recent systematic approaches based on molecular findings considered the Atractotrematidae to be the sister group of the Haploporidae [12, 14]. Unfortunately, the absence of complete ultrastructural study in the Haploporidae does not allow us to test their close relationship to the Atractotrematidae.

Regarding the position of the Haploporoidea, some molecular analyses have found (with very low support) the Opisthorchioidea as sister group of the Haploporoidea. Moreover, the “clade” Haploporoidea + Opisthorchioidea was found to be close to the Lepocreadioidea [14].

Spermatological studies available in the Opisthorchioidea concern the families Cryptogonimidae, Heterophyidae and Opisthorchiidae. The absence of submembranous electron-dense material or similar structures in all opisthorchioid species described so far is an ultrastructural argument that also could question the close affinity between Atractotrematidae and Opisthorchioidea.

However, comparison between mature spermatozoa of *A. sigani* and those reported in lepocreadioid species has revealed several similarities. They concern the morphology of anterior and posterior spermatozoon extremities, the presence of structure like submembranous electron-dense material, the association “external ornamentation + cortical microtubules”, the location of external ornamentation and the spine-like bodies. Although further studies are needed in the unexplored taxa, the ultrastructural characteristics described in *A. sigani* would suggest a close relationship between the Atractotrematidae and the Lepocreadioidea.

## Conclusion

The present study enlarges the data on ultrastructural studies in the Digenea providing, for the first time, spermatological characteristics in the Atractotrematidae. The mature spermatozoon of *A. sigani* exhibits, as do most digenean species, two axonemes with the 9 + “1” pattern of trepaxonematan platyhelminths, two bundles of parallel cortical microtubules, a nucleus, two mitochondria and granules of glycogen. Besides these ultrastructural features, the male gamete of *A. sigani* contains submembranous electron-dense material located in its anterior region, an association “external ornamentation + cortical microtubules” and spine-like bodies. The particular morphology of the anterior and posterior spermatozoon extremities and that of the submembranous electron-dense material distinguishes the spermatozoa of *A. sigani* from those described in other digenean species.

Regarding the possible phylogenetic implications of our ultrastructural analyses, the present study reveals several similarities between the mature spermatozoa of the atractotrematid *A. sigani* and those reported in lepocreadioid species. Herein, we suggest a close relationship between the Atractotrematidae and Lepocreadioidea rather than the Opisthorchioidea. However, additional ultrastructural and molecular studies are strongly needed in order to test our hypothesis and understand the relationship between the Atractotrematidae and other digenean families, particularly their most closely related family namely, the Haploporidae.

## Conflict of interest

The Editor-in-Chief of Parasite is one of the authors of this manuscript. COPE (Committee on Publication Ethics, http://publicationethics.org), to which Parasite adheres, advises special treatment in these cases. In this case, the peer-review process was handled by an Invited Editor, Dominique Vuitton.
